# Caveolae with GLP-1 and NMDA Receptors as Crossfire Points for the Innovative Treatment of Cognitive Dysfunction Associated with Neurodegenerative Diseases

**DOI:** 10.3390/molecules29163922

**Published:** 2024-08-20

**Authors:** Moeka Nakashima, Naoko Suga, Sayuri Yoshikawa, Satoru Matsuda

**Affiliations:** Department of Food Science and Nutrition, Nara Women’s University, Kita-Uoya Nishimachi, Nara 630-8506, Japanfyb83bh720fj@gmail.com (N.S.);

**Keywords:** caveolae, caveolin, glucagon-like peptide-1, NMDA, autophagy, mitophagy, cognitive dysfunction, Alzheimer’s disease

## Abstract

Some neurodegenerative diseases may be characterized by continuing behavioral and cognitive dysfunction that encompasses memory loss and/or apathy. Alzheimer’s disease is the most typical type of such neurodegenerative diseases that are characterized by deficits of cognition and alterations of behavior. Despite the huge efforts against Alzheimer’s disease, there has yet been no successful treatment for this disease. Interestingly, several possible risk genes for cognitive dysfunction are frequently expressed within brain cells, which may also be linked to cholesterol metabolism, lipid transport, exosomes, and/or caveolae formation, suggesting that caveolae may be a therapeutic target for cognitive dysfunctions. Interestingly, the modulation of autophagy/mitophagy with the alteration of glucagon-like peptide-1 (GLP-1) and N-methyl-d-aspartate (NMDA) receptor signaling may offer a novel approach to preventing and alleviating cognitive dysfunction. A paradigm showing that both GLP-1 and NMDA receptors at caveolae sites may be promising and crucial targets for the treatment of cognitive dysfunctions has been presented here, which may also be able to modify the progression of Alzheimer’s disease. This research direction may create the potential to move clinical care toward disease-modifying treatment strategies with maximal benefits for patients without detrimental adverse events for neurodegenerative diseases.

## 1. Introduction

Given the risk of worsening and changes in dementia, concern regarding cognitive dysfunctions has recently expanded. Cognitive dysfunctions are characterized by a reduction in the weight/volume of the brain due to brain cortical atrophy with a widening of the brain grooves. This brain atrophy predominantly encompasses the hippocampus. At this time, there is almost no treatment for cognitive dysfunctions [1,2]. Alzheimer’s disease is the most general type of cognitive dysfunction, affecting almost a quarter of people over 80 years old [1,2]. Alzheimer’s disease also accounts for about 60% of dementia cases, together with other dementias such as vascular dementia, Lewy body dementia, and frontotemporal dementia, as well as other neurodegenerative diseases. Alzheimer’s disease is also a chronic neurodegenerative disease, which is considered one of the most challenging medical problems with substantial financial and/or social costs. Discovery programs for the treatment of cognitive dysfunctions under clinical trials targeting inflammatory mediators, N-methyl-d-aspartate (NMDA) receptors, nicotinic acetylcholine receptors, secretase modulators, cholinergic system, tau, and glucagon-like peptide-1 (GLP-1) have been explored. Among them, the current drug used for the treatment of Alzheimer’s disease, memantine, an NMDA receptor antagonist, and acetylcholinesterase inhibitors may momentarily amend cognitive decline, even without the termination of dementia progression. Therefore, novel techniques are immediately required to considerably and safely improve cognitive impairment [3]. Further attention must be paid to the preclinical stages of dementia within research and/or medical areas. The identification of the modifiable risk factors and disease-modifying treatments for Alzheimer’s disease is urgently required to target the early or mild stages of cognitive dysfunction associated with neurodegenerative diseases [4,5].

Cholesterol is one of the most essential biomolecules in cellular physiology due to its involvement in several key biological processes. For example, cholesterol forms caveolae and/or lipid rafts that expedite the localization of several key molecules, including receptors and downstream enzymes in the cells of the central nervous system (CNS). Cholesterol also plays an important function in the plasticity of synapses [6]. Remarkably, the CNS is different from the other peripheral organs in terms of cholesterol metabolism and/or requirements [7]. The depletion of cholesterol from the plasma membranes of some neurons might lead to a complete loss of caveola structure, which may drastically impair their chemo-migratory responses [8]. The majority of risk genes for Alzheimer’s disease are commonly expressed within the brain microglia, which may be linked to cholesterol metabolism, lipid transport, endocytosis, exocytosis, and/or caveolae formation [9]. A strong genetic risk factor for Alzheimer’s disease may be the allelic variation of the *APOE* gene. However, the biochemical foundation for this risk profile is somewhat unclear. Through caveolae and cholesterol-rich lipid raft endocytic pathways within the CNS, it has been shown that some extracellular vesicles enter oligodendrocyte progenitor cells, exerting a pro-maturation effect on oligodendrocytes [10]. Accordingly, learning the roles of cellular membrane biophysics with neuronal/microglial functions should improve our understanding of the pathology of the cognitive dysfunction associated with neurodegenerative diseases.

## 2. Caveolae

**Figure 1:**
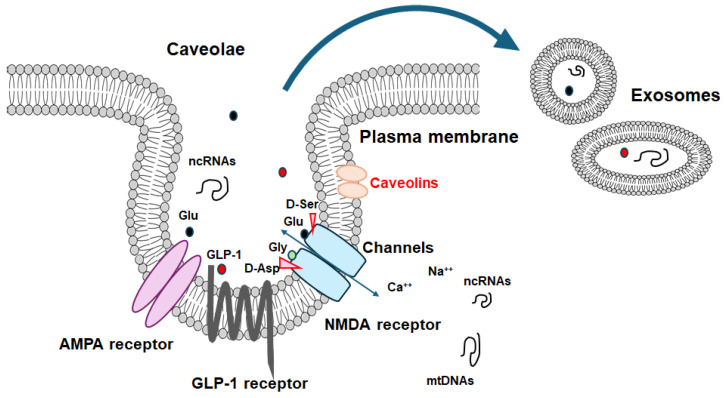
An illustrative representation of caveolae associated with caveolin proteins. Caveolae may be a kind of platform for various signaling molecules including GLP-1 and NMDA receptors as well as even for the development of certain exosomes, which may be interrelated to the scaffold domain of caveolins. Note that some critical pathways for the development of various disease-related signaling have been omitted for clarity. Abbreviation: GLP-1, glucagon-like peptide-1; NMDA, N-methyl-d-aspartate.

Caveolae are approximately 100 nm flask-shaped invaginations of the plasma membrane that show the presence of caveolin proteins [11]. Caveolae may also hold several signaling proteins, including various transmembrane receptors, through their interaction with some caveolins, which have been theorized to act as crucial hubs for facilitating intracellular signal transduction [12] (Figure 1). Therefore, caveolae might be involved in the formation of synapses and their plasticity. For example, neuron-targeted caveolin-1 may improve NMDA- and/or BDNF-mediated signaling, which could increase the growth of dendritic cells [13]. Consequently, mechanical changes in hippocampal neurons may be associated with enhancements in the hippocampal functions of learning and/or memory [14]. In addition, a different study has revealed that caveolin-1 can interact with the function of several metabotropic glutamate receptors in the hippocampus, assisting their expression [15]. Caveolin-1 might also regulate the transport of metabotropic glutamate receptors to the membrane surface, which may be responsible for the synaptic facilitation in the formation of long-term potentiation (LTP) [16]. Caveolin-1 may also be involved in the endocytosis of AMPA receptors in the hippocampus, which might reverse metabotropic glutamate receptor-related long-term depression (LTD) [17]. Conversely, the overexpression of caveolin-1 could promote the endocytosis of AMPA receptors from caveolae-like structures within the neurons of the hippocampus, which may assist the contribution of caveolin-1 to receptor trafficking [18]. Interestingly, it has been revealed that AMPA receptor trafficking, possibly mediated by caveolin-1, could improve primary behavioral disorders [17,19]. Therefore, caveolin-1 might be important for the augmentation of metabotropic-glutamate-receptor-related LTD, and for further mediating AMPA receptor trafficking.

In general, the key protein of caveolae is the caveolin-1 that is a 23 kDa of membrane protein with a hairpin-like structure ubiquitously expressed in various tissue cells, while caveolin-3 is extremely expressed in striated muscle cells [20]. Caveolin-1 and caveolin-2 are co-expressed in various cell types, including epithelial cells, endothelial cells, and/or fibroblasts. In contrast, caveolin-3 expression is basically limited to skeletal muscle cells as well as cardiac myocytes. Their functional role in cell types expressing different isoforms have yet to be identified precisely. The amino- and carboxy-terminal domains are preoccupied with the cytoplasm, whereas the hairpin loop is in transmembrane zone. In addition to create caveolae structure through the hetero-oligomeric complex with caveolin-2, the juxtamembrane region in the amino-terminus of the caveolin-1 may specially work as a scaffold protein, associating with a variety of signaling molecules including G-proteins [21,22]. Moreover, caveolin-1 can significantly cooperate with insulin receptors, maintaining a role in energy/metabolic regulation [23]. Interestingly, it has been revealed that caveolin-1 could endorse the structural plasticity of neurons as well as neurogenesis in the brain, suggesting that caveolae may be a fruitful therapeutic target for considering cognitive dysfunctions [24]. In addition, caveolin-1 has been a possible therapeutic target for a diabetes-induced cognitive dysfunction [25]. Remarkably, the alteration of caveolin-1 could induce memory deficits that take after the neurological phenotype of Alzheimer’s disease [26]. Caveolin-1 can physically associate with the mature amyloid precursor protein [27]. The roles of caveolins at caveolae might affect the neurological condition conceivably through an anti-oxidative stress-dependent mechanism in the process of neurodegeneration [26,28,29]. It has been shown that caveolin-1 may be a novel candidate with neuroprotective and/or anti-oxidative effects through modulating neuronal ferroptosis-mediated mitochondrial homeostasis [30]. Further studies are indispensable in order to evaluate the precise effect of caveolae dysfunction for the cascade of cognitive dysfunction associated with neurodegenerative diseases.

## 3. Two Relevant Key Receptors to Cognition in Caveolae

**Figure 2:**
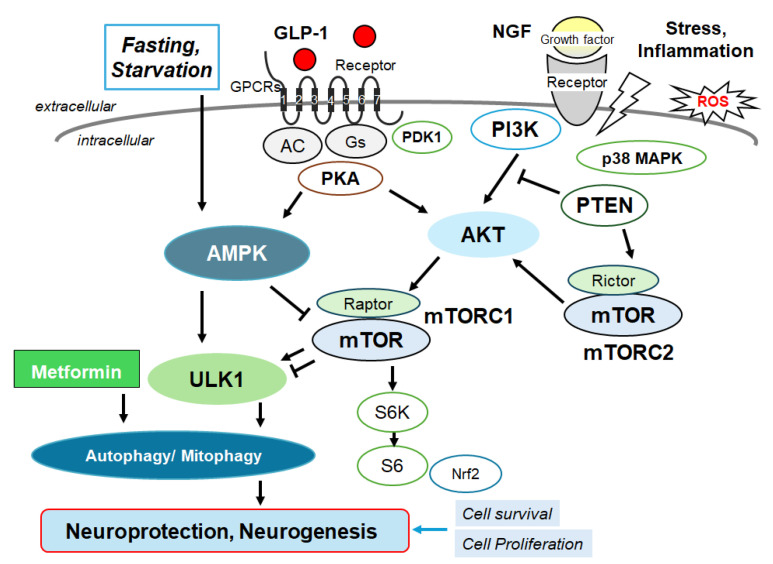
Several modulator molecules linked to the signaling of GLP-1 receptor within the PI3K/AKT/mTOR pathway are demonstrated. Example molecules known to act on an AMPK/mTOR pathway are also shown. The activation of GLP-1 receptor results in the stimulation of adenylyl cyclase (AC) that transforms adenosine triphosphate (ATP) into 3-5-cyclic adenosine monophosphate (cAMP), which then activates the cAMP-dependent protein kinase (PKA) as well as the following AMPK for the modification of autophagy/mitophagy. Some example factors including fasting, starvation, and/or metformin known to act on the autophagy/mitophagy signaling are also shown. Note that some critical pathways such as that for insulin induction have been omitted for clarity. Arrowhead means stimulation whereas hammerhead represents inhibition. Abbreviation: GLP-1, glucagon-like peptide-1; AMPK, adenosine monophosphate-activated protein kinase; mTOR, mammalian/mechanistic target of rapamycin; PI3K, phosphoinositide-3 kinase; PKA, protein kinase A; PTEN, phosphatase and tensin homologue deleted on chromosome 10.

The activation of GLP-1 receptors and their receptor internalization may result in the intracellular signaling that is regulated by the availability of downstream molecules. In addition, it has been shown that the GLP-1 receptor co-localizes with the caveolin-1 at caveolae, which may be involved in controlling the receptor activity by assembling signaling complexes with the receptor trafficking [31]. In fact, the sequence of GLP-1 receptor contains a classical caveolin-1 binding motif within the second intracellular loop [32], by which the GLP-1 receptor can directly associate with the caveolin-1 [33]. This interaction may be required for the internalization of receptors in caveolae and also for the removal of the GLP-1 receptor [34,35]. On the contrary, the GLP-1 receptor is not internalized even after the agonist stimulation in cells expressing the P132L-altered caveolin-1, a mutated form of the caveolin-1, which may result in misfolded oligomers accumulated within the Golgi complex [36]. There are several reports that caveolin-1 mutants, P132L and/or Y14F, may be dominant negative regulators of caveolin-1. The activated standard GLP-1 receptor generally undergoes agonist-mediated endocytosis, which may lead to recycling the receptor either back to the plasma membrane or to the degradative autophagosome pathway [37]. Since caveolin-1 may be required for the internalization of the GLP-1 receptor after an agonist stimulation, it is plausible that caveolin-1 might also affect the fate of the GLP-1 receptor, determining either the degradation or recycling of the GLP-1 receptor [38]. Therefore, the association between the GLP-1 receptor and caveolin-1 is crucial not only for receptor trafficking, but also for the instigation of various intracellular signaling pathways. Remarkably, the GLP-1 receptors in the hippocampus of the brain are also involved in the modulation of NMDA receptor activity through the modulation of the cAMP-response element binding protein (CREB) [39]. In addition, GLP-1 analogues may also be useful for the treatment of Alzheimer’s disease, because they have been linked to neuroprotective and/or anti-inflammatory properties [40]. The modulation of GLP-1 activity could affect the aggregation of amyloid-beta in the brain of Alzheimer’s disease [40]. The GLP-1 receptor agonists have been shown potentially to have favorable actions on the brain with several neurological deficits via the phosphoinositide-3 kinase (PI3K)/AKT/mTOR signaling pathway [41] (Figure 2). It has also been shown that downstream ionotropic glutamate receptors such as NMDA receptor signaling in the hippocampus may be required for the GLP-1-receptor-mediated behaviors, suggesting that the signaling from GLP-1 receptors to a NMDA-receptor pathway may be at least modulating a behavior in the realm of neuronal control. [42]. NMDA receptors are kinds of ionic glutamine receptors involved in brain development and/or functions such as learning and memory formation. Synaptic NMDA receptors are indeed important for synaptic plasticity and neuronal survival, which might also play an indispensable role in memory and learning [43]. This NMDA receptor may be responsible for mediating LTP and maintaining brain health. Physiological precise functions of NMDA receptors may be shaped by their subunit composition within the CNS [44,45]. It is worth noting that recent studies in mice have detected a reduction in the expression of the NMDA receptor to the dendritic spine, leading to the formation of extrasynapse, which is associated with advanced neuronal cell death and accelerated age-related cognitive decline [46]. In addition, targeting the NMDA receptor has been proposed to be therapeutically advantageous in addressing various neurodegenerative disorders. Up to now, a great deal of the related targets have been explored [47]. Illuminating a novel functional harvest of the GLP-1 receptor and NMDA receptor in the neural connectivity of brain hippocampus, this may enlighten novel neurological targets for the treatment of cognitive dysfunctions. However, translating studies for the agonists/antagonists of these receptors into fruitful clinical medications has been a hard mission. This is principally owing to the complexity of receptor signaling, as their functions might profoundly be associated with various fundamental functions of the brain. The abnormality either of the hypofunction or hyperfunction of signaling may be harmful to the whole brain health [48]. Under physiological conditions, the receptor signaling may firmly be operated for ensuring optimal function in the CNS. A difference from this optimum might be deleterious. For example, the use of NMDA receptor agonists/antagonists in various neurodegenerative disorders has not yet to produce the satisfactory consequence, suggesting an inadequate understanding of the receptor’s function in the brain [49]. In addition, some of these agonists/antagonists have been obvious by detrimental adverse effects such as schizophrenia-like psychological effects [50]. The significance of glutamate-induced excito-toxic neuronal cell death in the pathogenesis of neurodegenerative disorders has been well-recognized. Although memantine and amantadine are clinically accessible, they are not without adverse effects at all. Similarly, ifenprodil, a selective allosteric modulator targeting a NMDA receptor, has exhibited positive results with good profiles of small adverse effects, only suggesting symptomatic improvement. A better understanding of the mechanism of agonists/antagonists to GLP-1 and NMDA receptors is anticipated.

## 4. Autophagy/Mitophagy

It is of great importance that we identify effective interventions that could delay or prevent cognitive dysfunctions. In view of the fact that the hyperactivation of mTOR in patients with Alzheimer’s disease may impair autophagy, contributing to the accumulation of plaques and tangles, the dysregulation of autophagy is a key pathological feature of Alzheimer’s disease [51]. In this regard, it has been shown that the p62 molecule, an autophagy receptor, is a multifunctional protein that has been associated in the pathology of Alzheimer’s disease due to its ability to attach with neurofibrillary tangles for degradation [52]. Additionally, the mitochondrial dysfunction with the increase in damaged mitochondria, an impairment of mitophagy/autophagy, has been well-known to contribute to cognitive dysfunctions [53]. Therefore, improved autophagy and/or mitophagy could reduce the pathology of Alzheimer’s disease [54]. Interestingly, a promising applicant material for this is the disaccharide trehalose, which has shown the effectiveness in inhibiting experimental neurodegeneration in several models of Alzheimer’s disease [55]. Some mechanisms of therapeutic action have been identified, which suggests that trehalose can regulate autophagy by encouraging rapid lysosomal enlargement and membrane permeabilization correlating with the calcium-dependent calcineurin activation [56]. By stimulating autophagy/mitophagy, this substance could consequently enhance the removal of cytotoxic proteins including amyloid-β aggregates as well as damaged mitochondria, suggesting that autophagy/mitophagy could possess considerable therapeutic activity [57,58]. Autophagosomal activation by the treatment of trehalose could also remove the cytotoxic properties of things including hyper-phosphorylated tau [58,59]. Notably, the experimental alteration of autophagy/mitophagy could commonly hamper the neuroprotective effect of trehalose [60]. In addition, it has been revealed that the dose-dependent effects of appropriate autophagy in the hippocampus and/or frontal cortex could improve cognitive impairment as well as behavioral disturbances [61]. Now, autophagy/mitophagy has been identified as a strategic player and/or target in the pathology of cognitive dysfunctions or neurotoxicity.

**Figure 3:**
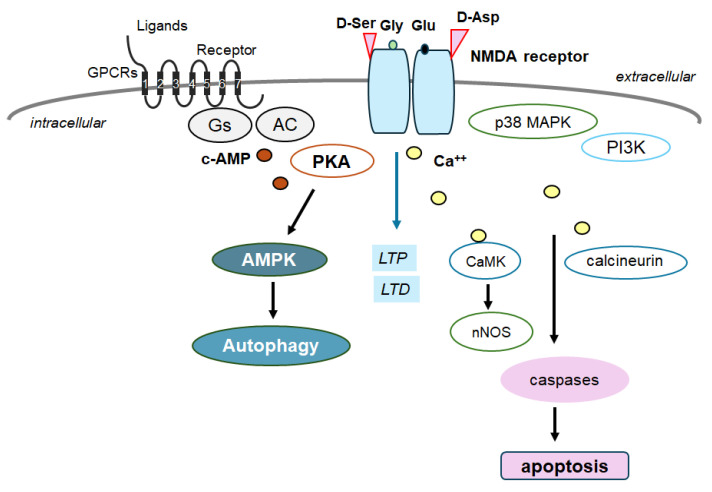
Schematic depiction of NMDA receptor signaling pathway along with a GTP-binding protein-coupled receptor (GPCR) signaling pathway for autophagy/mitophagy. NMDA receptors may require the binding of several amino acid molecules of glycine, glutamate, and/or aspartate, which could modulate the receptor function. For example, glutamate is in the glutamate-binding site and glycine is in the glycine-binding site within the NMDA receptor subunit. The stimulation of NMDA receptors may allow Ca^2+^ entry into the cytoplasm of neurons, which may contribute to long-term potentiation (LTP) or long-term depression (LTD) as well as to a phase of excito-toxicity and/or apoptosis. Stimulatory effects are indicated with arrows.

**Figure 4:**
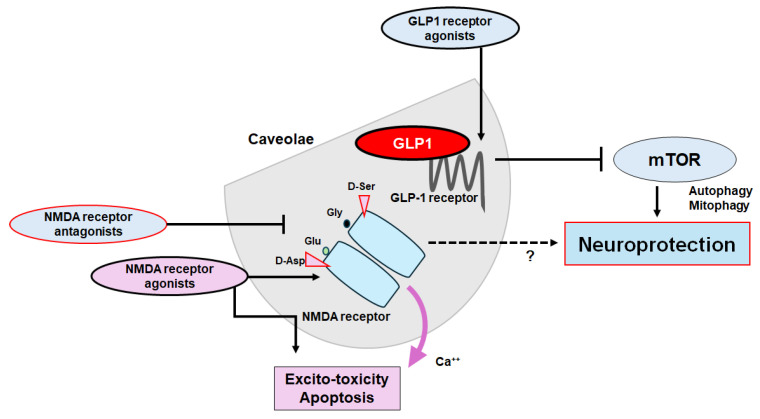
The plausible tactics for neuroprotection and/or against cognitive impairment have been shown. The ideal approach might be to utilize agonists of GLP-1 receptors and/or antagonists of NMDA receptors to halt the neuron-damaging process for neuroprotection. Note that several important activities such as inflammatory reaction, autophagy initiation, and reactive oxygen species (ROS) production have been omitted for clarity. Stimulatory effects are indicated with arrows; inhibitory effects with a line ending in a hammerhead. Broken lines indicate possible mechanism of action. “?” means author speculation.

In general, autophagy/mitophagy is developed to clear misfolded proteins and damaged mitochondria, thereby supporting cellular homeostasis and survival [62], which may be regulated by the adenosine-monophosphate-activated protein kinase (AMPK) and mammalian/mechanistic target of rapamycin (mTOR) cascade [63]. Remarkably, the activation of the GLP-1 receptor could expedite the autophagy in the relevant signaling pathways [64] (Figure 2). Interventions of the GLP-1-mediated modulation of autophagy/mitophagy might be applied in clinical practice to reduce the incidence of type 2 diabetes mellitus for the protection of islet beta-cells [65]. It is known that amyloid-β could interact with the neuronal NMDA receptor, resulting in the excessive entry of calcium, thereby causing excito-toxicity for neuronal cells [66] (Figure 3). Memantine, an FDA-approved drug usually prescribed for patients with Alzheimer’s disease, is a non-competitive inhibitor of the NMDA receptor, which could work in the regulation of calcium entry into cytoplasm [67]. Additionally, memantine has also been shown to stimulate autophagy, reducing the accumulation of amyloid-β with the amended survival of neurons [68,69]. Another antagonist for the NMDA receptor with calcium channel-blocking might also be shown to slowdown the cognitive decline [70]. Because the modulation of NMDA receptor activity could change the excitability of neuronal routes, however, slight differences in the mechanisms of action of NMDA receptor antagonists could fervently impact their clinical effects [70] (Figure 4).

## 5. Clinical Translation

As for clinical translation, the effectiveness with less adverse events would be critical when applied in any medical science. The phenomenon of excito-toxicity with glutamate stimulation may have guided us to the discovery of new molecules that could be employed in treating neurodegenerative diseases by obstructing NMDA receptors. Among the NMDA receptor inhibitors, memantine has been permitted for the treatment of Alzheimer’s disease and/or dementia; however, no other inhibitor of NMDA receptors has been approved for clinical trials [71]. Memantine, an NMDA receptor channel blocker, could prevent glutamine hyper-activity in Alzheimer’s disease by modulating autophagy, which has been used as well for the treatment of mild cognitive dysfunction associated with neurodegenerative diseases [72,73]. Memantine could bind the NMDA receptor within the entrance of the ion channel, which may help to close the ion channel gate, actually blocking ion permeation [73]. The excess activation of superfluous NMDA receptors could bring about the death of glial cells/neurons that is deliberated as a relevant factor in the development of Alzheimer’s disease, which could, at least in part, be avoided by memantine [74]. Another receptor antagonist ifenprodil has been found to be an effective negative modulator that could also inhibit NMDA receptors including the GluN2B subunit [75]. The arrangement of memantine with ifenprodil or a selective serotonin reuptake inhibitor (SSRI) has been more proficient in improving cognitive significances [75,76]. Interestingly, it has been shown that citalopram, an SSRI, could also activate autophagy/mitophagy in patients with Alzheimer’s disease [77]. On the other hand, GLP-1 receptor agonists may also prevent the neurotoxicity associated with Alzheimer’s disease, while presently encouraged for the treatment of type 2 diabetes or obesity. For example, one of the GLP-1 receptor agonists, semaglutide, has been clinically studied for the reduction in neuroinflammation as well as amyloid-β and/or cortical tau protein in the brain with Alzheimer’s disease [78]. Exenatide, another GLP-1 receptor agonist, may be an available therapeutic agent also presently used in the treatment of diabetes or obesity, which might have additional and favorable effects on cognitive function in human patients [79]. Furthermore, the impact of GLP-1 from dipeptidyl peptidase-4 inhibitors has also been evaluated on cognitive function in patients with type 2 diabetes mellitus [80].

Parenthetically, preceding investigations have emphasized that spermidine has a favorable ability to initiate the process of disappearing amyloid-beta plaques by the mechanism of autophagy [81]. In addition, polyphenols such as anthocyanins could also amend the memory, learning, and cognitive function by modifying autophagy [82]. Interestingly, mild fasting might also stimulate autophagy for the beneficial effects in brain function [83]. In line with this, a recent study has suggested that a form of recurrent mild fasting within a restricted time frame may contribute to the treatment of neurodegenerative diseases, which might improve autophagy, decrease oxidative stress, and expand cognitive function [84]. Similarly, it has been shown that a diet restricted to the circadian rhythm may stimulate the autophagy, producing an increased level of brain-derived neurotrophic factor (BDNF) in the forebrain area, and increasing synaptic plasticity, thus enhancing cognitive function [85]. In these ways, clinical studies have currently shown that appropriate autophagy/mitophagy can significantly improve cognitive function, although the mechanisms are not completely understood.

## 6. Future Directions and Conclusions

Dementia, cognitive disorders, and/or depression are now notorious, owing to several limitations in therapy. Strategies that slow or prevent the clinical progression of these disorders have mostly remained elusive, until recently. Therefore, further investigations involving validated tools for therapy are required. Many competitive or noncompetitive agonists/antagonists of GLP-1 and/or NMDA receptors might have been demonstrated to have undesirable adverse effects, which are connected to the receptor-binding affinity and/or allosteric modulation of receptor function. Through the NMDA ionotropic receptor, certain amino acids could modify intracellular Ca^2+^ dynamics, allowing the cell proliferation and/or apoptosis in neurulation. Glutamate synthesis depends on mitochondrial enzymes such as glutaminase 1 (GLS1) that is widely expressed in brain, which may contribute to neurogenic processes [86]. Interestingly, it has been shown that NMDA receptor antagonism with GLP-1 receptor agonism may effectively reverse the hyper-glycemia, dyslipidemia, and/or obesity in the animal model of metabolic diseases [87]. These modulators may offer potential even in addressing various psychiatric and/or neurodegenerative disorders. Therefore, a more comprehensive exploration of the structure and/or function of these receptors would have led to the development of some specific modulators with satisfactory side-effect profiles. For example, a pharmacological NMDA receptor blockade could attenuate the food intake decrease resulting from the stimulation of astrocytes, which could also modulate the fasting or anorexic effect of GLP-1 [88]. As food intake behavior may be under the strict control of the CNS, the signaling actions of the NMDA receptor with the GLP-1 receptor might be encouraging for the safe modulation of the CNS. Nevertheless, you cannot see the forest for the trees. The ideal approach would be to utilize agonists of GLP-1 receptors and/or antagonists of NMDA receptors to halt the neuron-damaging process for neuroprotection. Currently, these approaches for the treatment of cognitive dysfunction associated with neurodegenerative diseases do have limitations due to their detrimental adverse effects. However, these limitations might have given researchers further insight on how to augment the efficacy in order to enable the precise adjustment of autophagy/mitophagy and enhance the crossfire of therapeutics at the caveolae site (Figure 4). The use of biomarkers that indicate pathological alterations suggesting the development of cognitive dysfunctions may significantly contribute not only to the effort to identify the disease as early as feasible but also to the evaluation of the precise therapeutic efficacy during the disease process with the treatment. Probing the minimal essential dose for effective and safe treatment is necessary for real and actual therapy. Alongside this, clinical trials should evaluate the impact of appropriate biomarkers that could theoretically detect the crawl of the disease progression by the treatment.

Exosomes are a kind of extracellular vesicles released upon exocytosis, characterized by a bilayer membrane structure (Figure 1). Exosomes in the CNS could transmit messages across the brain via the cerebrospinal fluid (CSF) [89]. Exosomes, containing specific cargoes such as lipids, proteins, and nucleic acid molecules including non-coding RNAs or DNA and other bioactive compounds, are transported from donor cells to distant recipient cells through mechanisms like endocytosis/phagocytosis, exocytosis, and/or plasma membrane fusion, facilitating an information transmission and/or a material movement. Therefore, exosomes may participate in intricate cell-to-cell communication, which may play a key role in the development of several tissues/organs including the CNS. Interestingly, it has been shown that exosomes with mitochondrial DNA may possess potential as an indicator of cognitive consequences [90]. Recently, the increased study of exosomes has led to increasing attention on their involvement in neurodegenerative disorders including Alzheimer’s disease. While therapeutic treatments with nanotechnology might currently be effective in treating cognitive dysfunctions, those exosomes should be applied to the evaluation of the precise therapeutic efficacy during the disease progression/suppression with treatments. Again, there is an urgent need for diagnostics and/or therapeutic options against cognitive dysfunction associated with neurodegenerative diseases. Additionally, certain exosomes may have a key role in Alzheimer’s disease by contributing to the alleviation of the pathological progression of the disease [91]. For example, some exosomes have been detected to regulate specific microRNAs including miRNA-17-5p, miRNA-21, and miRNA-126-3p, encouraging their therapeutic potential [91]. By modulating autophagy, some exosomes may potentially boost neurogenesis, contributing to the improvement of learning and/or memory, decreasing Aβ accumulation, and thus opposing Alzheimer’s disease [92]. In fact, a novel mechanism has been recognized linking some exosomes and synaptic susceptibility for the innovative treatment of cognitive dysfunctions [93]. Since exosomes are typically derived from the invagination of cell plasma membranes, caveolae may be related to exosome biogenesis [94]. It is worth mentioning that the brain can discharge several exosomes to communicate with peripheral along with distant cells/organs, which might also assist in the homeostasis of brain cognitive action.

Caveolae are structural cholesterol-rich microdomains involved in effective signal transduction and the modulation of physiological processes of the plasma membrane, which may also be involved in a wide range of neurodegenerative diseases such as Alzheimer’s disease. In addition, it has been shown that significant functions of caveolae may be involved in various cell types of the CNS including astrocytes, microglia, oligodendrocytes, and/or neurons [95]. This pancellular presence of caveolae in the CNS requires a better consideration of their functional roles in each cell type. Based on the available evidence and/or reconstitution of caveolae with the alteration of GLP-1 and NMDA receptor signaling, this could further be a promising treatment strategy for a wide range of cognitive dysfunction associated with various neurodegenerative diseases.

## Data Availability

Not applicable.

## Abbreviations

AMPK	Adenosine-monophosphate-activated protein kinase
BDNF	brain-derived neurotrophic factor
CSF	cerebrospinal fluid
CNS	central nervous system
GLS1	glutaminase 1
GLP-1	glucagon-like peptide-1
CREB	cAMP-response element binding protein
LTD	long-term depression
LTP	long-term potentiation
mTOR	mammalian/mechanistic target of rapamycin
NMDA	N-methyl-d-aspartate
ROS	reactive oxygen species
SSRI	selective serotonin reuptake inhibitor
